# Self-Assembly of Ultrafine Fibers with Micropores via Cryogenic Electrospinning and Its Potential Application in Esophagus Repair

**DOI:** 10.3390/polym14091924

**Published:** 2022-05-09

**Authors:** Wenqing Tian, Xinghuang Liu, Xianglin Zhang, Tao Bai, Bin Wu

**Affiliations:** 1 State Key Laboratory of Materials Processing and Die & Mould Technology, School of Materials Science and Engineering, Huazhong University of Science and Technology, Wuhan 430074, China; tianwenqing13@163.com (W.T.); hust_zxl@mail.hust.edu.cn (X.Z.); 2 Division of Gastroenterology, Union Hospital, Tongji Medical College, Huazhong University of Science and Technology, Wuhan 430022, China; xh_liu@hust.edu.cn (X.L.); drbaitao@126.com (T.B.)

**Keywords:** addictive manufacturing, electrospinning, thin film, esophageal tissue engineering

## Abstract

Electrospinning (e-spinning) has been widely applied to fabricate flat films accumulated by microfibers for tissue engineering. In order to acquire an uneven surface morphology, two methods have been applied traditionally. The first uses a designed receiving substrate, which is stable, but suppresses the flexibility. The second uses dual solvents to achieve bimodal distribution of the fiber diameter. However, the bimodal fiber diameter causes inhomogeneity. To solve these challenges, cryogenic electrospinning, using a flat substrate and a single solvent, was performed in this study to obtain uneven films. By applying a low temperature to the flat receiving substrate, uneven e-spun films with wall-like structures were achieved through the self-assembly of uniform filaments. In addition, the wall-like structures enhanced the mechanical properties of the e-spun films. Moreover, the cryogenic e-spinning produced micropores on the fiber surface, which have the potential to promote esophageal epithelial cell adhesion, proliferation and differentiation.

## 1. Introduction

Electrospinning (e-spinning) is a commonly used technique to fabricate flat films for tissue engineering, providing an extracellular matrix for cell adhesion, proliferation and differentiation [1,2]. However, cells are likely to attach to uneven, rather than flat, surfaces. In order to obtain uneven, honeycomb-like structures, a predesigned template was utilized to trigger the directional deposition of e-spun fibers [3,4]. In this way, fibers were deposited preferably on the solid region of the templates, instead of on the hollow regions. This uneven architecture has proved to be beneficial for cell proliferation and tissue regeneration [5,6]. However, the utilization of a structured template increases the complexity and cost. Moreover, the shape of the obtained e-spun structure is fixed on the template shape, which is not flexible.

To resolve this challenge, researchers have explored e-spinning self-assembly as an alternative template-free method to acquire honeycomb-patterned nanofibrous structures [6,7]. Self-assembly in electrospinning refers to the spontaneous bottom-up stack of e-spun fibers on a certain area, to form a clearly uneven structure [8]. The mechanism aims to realize the inhomogeneous distribution of residual charge in the filaments by attracting flying fiber to cumulate on a multi-charge area of the membrane, and ultimately causing the preferential accumulation of fibers [7,9]. For example, Yao [9] fabricated a gradient honeycomb nanofibrous mesh through e-spinning self-assembly without a template, and was able to control the pore diameter by altering the working distance. Hanumantharao [10] used e-spinning self-assembly to form a pattern with a long-range order. However, up to now, a bimodal fiber diameter has been necessary for e-spinning self-assembly, which indicates that self-assembly is not possible when the diameter of the e-spun filament is uniform [4,10]. For example, bead-like parts along the fiber would trigger self-assembly, since the beads act as point charges on the deposited region, attracting areal fibers and forming a fiber cluster. Therefore, it is appealing to investigate the possibility of homogeneous fiber self-assembly, which would result in a defined fiber surface quality and more precise regulation [11,12].

The existing self-assembly of e-spinning is based on the application of dissolving one solute in two different solvents, achieving inhomogeneity of the solution, hence causing charge accumulation. However, the inhomogeneity of the solution also inevitably causes inhomogeneity of the e-spun filament diameter. To obtain a homogeneous e-spun filament diameter, a cryogenic condition and the single solvent system can be applied during e-spinning. Cryogenic printing can be used to achieve phase separation during fiber formation, which causes the charge to accumulate and the micropores on fibers to form [13,14]. In this study, we combined cryogenic printing with e-spinning to induce the self-assembly of homogeneous PCL filaments without any templates. Macro uneven film structures of e-spun film were achieved. Furthermore, the micropores on the fiber surface provided hierarchical structures.

## 2. Materials and Methods

### 2.1. Materials

Polycaprolactone (PCL) pellets (molecular weight ~80 kDa) were purchased from Perstorp UK Ltd. (Warrington, UK), and glacial acid (GAC) was obtained from Sinopharm Chemical Reagent Co. Ltd. (Shanghai, China).

### 2.2. Cryogenic E-Spinning

The PCL pellets were directly dissolved in GAC to prepare the solution. After stirring for 3 h at 60 °C, the solution was loaded into a 10 mL injector. The pushing rate of the rotating pump was set at 0.04 mm/min. A high voltage was applied between the needle and the copper tablet. The square copper was set on the surface of the cooling plate and linked to the negative charge of the potential transformer. The temperature of the cooling plate could be set as low as −20 °C. A thin silicon wafer was used to collect the e-spun fiber mesh. The mesh could be easily peeled off after deposition. The working distance between the needle and the collector could be set from 0 to 30 cm. Four groups of fiber working distances, 15 cm, 13 cm, 9 cm and 8.5 cm, were used in the experiment. The voltage used during the experiment was 6.0 ± 0.3 kV to ensure the shape and stability of the Taylor cone. The humidity was controlled at a steady value during the experiment. A solution concentration of 30 wt.% was prepared for experimental use. The obtained mesh was stored in a refrigerator at −20 °C for 24 h, and then lyophilized for another 24 h. Key processing parameters, including receiving temperature, humidity and working distance, were investigated to obtain the optimal morphology.

### 2.3. Cell Culture

Immortalized human esophageal epithelial SHEE cells were purchased from Wuhan CRK Pharma Company. The cells were cultured in Roswell Park Memorial Institute essential medium (RPMI-1640; Gibco, Waltham, MA, USA), and supplemented with 10% (*v/v*) fetal bovine serum (Gibco, Waltham, MA, USA) and 1% (*v/v*) antibiotic/antimycotic solution (Gibco; final concentrations of 10000 units/mL penicillin G, 100 mg/mL streptomycin sulfate and 25 µg/mL amphotericin B) at 37 °C with 5% CO_2_ in a humidified atmosphere. The cells were put into T25 flasks and were cultured to around 80% confluence before being harvested using trypsin/EDTA (Sigma-Aldrich, St. Louis, MO, USA). The cell culture medium was replaced every 2 days. Generally, SHEE cells were seeded at a concentration of 1 × 10^5^ cells/mL, respectively. In this experiment, 1 mL of cell suspension was applied to the scaffold with a diameter of 12 mm. Before seeding the cells, the surface of the electrospun membrane needed to be coated with collagen. First, the membrane surface was washed three times with sterile PBS. It was then placed on a sterile operating table to dry naturally and illuminated with ultraviolet light for 1 h. The sterilized electrospun membranes were placed in a type IV collagen solution (the type IV collagen solution concentration was 1 ug/mL, while the dosage was 1 mL/cm^2^) at 37 °C overnight (12–24 h). Afterwards, the electrospun membranes and collagen suspension were placed on a sterile operating table to air dry. The dried electrospun membranes were washed three times with a sterile PBS solution for cell seeding.

### 2.4. Assessment of Cell Viability

Cell viability was detected using a Calcein/PI Cell Viability/Cytotoxicity Assay Kit (Beyotime, China) in accordance with the manufacturer’s instructions. Briefly, scaffolds containing living cells were incubated with calcein/PI dye solution (1:1000, 500 μL for a round piece of scaffold) for 1 h. The dead cells, which had PI-labeled red nuclei, and the viable cells, which had calcein-labeled green nuclei, were counted under a fluorescent microscope.

### 2.5. Characterization 

The surface morphology and cell morphology of cryogenic e-spun membranes were observed using a scanning electron microscope (JSM-7600F). When observing the cell morphology, the samples were prepared by rinsing the membranes three times with PBS, fixing in a 4% paraformaldehyde solution for 30 min at room temperature, and rinsing again three times. Afterwards, the membranes were dehydrated with gradient ethanol (10, 20, 30, 40, 50, 60, 70, 80, 90 and 100%) for 30 min and finally freeze-dried for 24 h. All samples were gold-coated for 360 s and observed under SEM. An X-ray diffraction (X’pert3 powder) study was performed. Three kinds of samples with characteristic morphologies were tested here, including non-woven flat membranes, uneven membranes with a honeycomb structure, and hierarchically structured fibrous membranes with both a macroscopic honeycomb structure and a fibrous surface porous structure. An E1000 All-Electric Dynamic Test Instrument was used to measure the mechanical properties of the e-spun mesh. In the mechanical properties test, the membrane along the orientation of the wall was cut into a rectangle with a length of 2 cm and a width of 1 cm. Variation in the height direction of the e-spun film was observed using an Infinite Focus Optical 3D surface metrology (IFM G5) tool.

## 3. Results and Discussion

**Figure 1:**
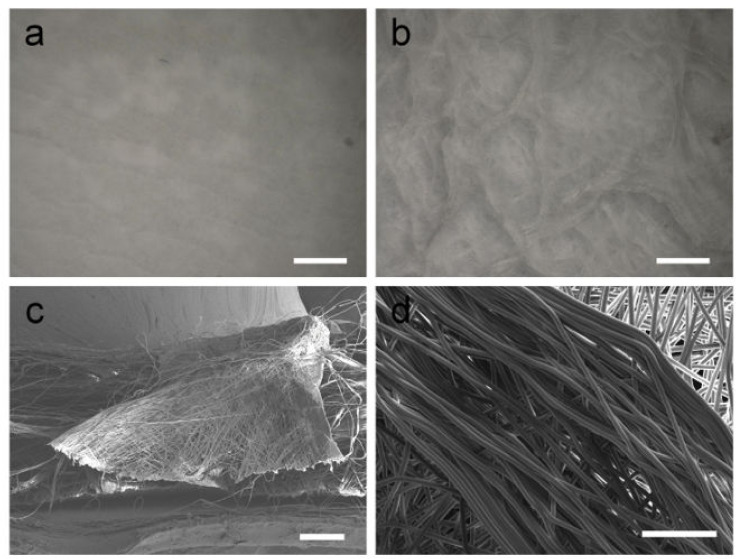
Surface macrostructure of electrospun films when the receiving temperature is (**a**) 23 °C and (**b**) −10 °C (the humidity is 50%, and the collecting distance between the needle and collector is 15 cm); (**c**) section image of wall-like structures; (**d**) top view of wall-like structures. Scale bars are 1 mm (**a**,**b**), 200 μm (**c**) and 50 μm (**d**).

**Figure 2:**
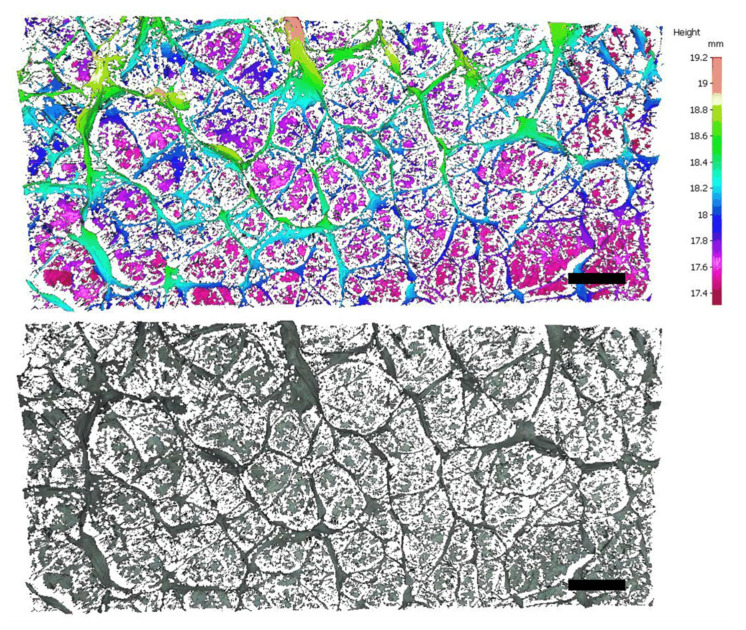
Surface profiles of uneven honeycomb films. Scale bars are 2 mm.

The selective deposition of fibers is key to obtaining honeycomb-like patterns under template-free electrospinning conditions. When no self-assembly occurs, the fibers obtained are not aligned and have no interwoven structures. The surface of the PCL fiber membrane obtained by electrospinning at room temperature is flat, and there is no self-assembly phenomenon (Figure 1a). However, when the temperature of the receiving plate was set at −10 °C, a fibrous membrane with an uneven surface and a macroscopic honeycomb structure was obtained using cryogenic electrospinning (Figure 1b). The structure at the bulge is shaped similarly to a wall. The results obtained by observing the wall-like structures via SEM are shown in Figure 1c,d. It can be observed from the section image that the filaments on the wall-like structure are stacked from the bottom to the top, forming a wall structure with a certain height. At the gathering region, it can be clearly observed that the fibers are arranged in the same orientation, and the parallel fibers form wall-like structures. A self-assembled fiber membrane with a macroscopic honeycomb structure can be obtained using cryogenic electrospinning technology. Under the combined action of an electrostatic force and a cryogenic receiving platform, the fibers in some areas are highly parallel and form a wall-like structure. In addition, the cryogenic receiving platform is an indispensable condition for the self-assembly phenomenon of the PCL/GAC solution. The distribution of a honeycomb structure on an uneven membrane surface over a large area was observed by the Infinite Focus Optical 3D surface metrology tool; the maximum height of the accumulated e-spun fibers was found to be 2.13 mm (Figure 2). The honeycomb structures fabricated by cryogenic electrospinning display obvious differences in the height direction, and the gradient is large.

**Figure 3:**
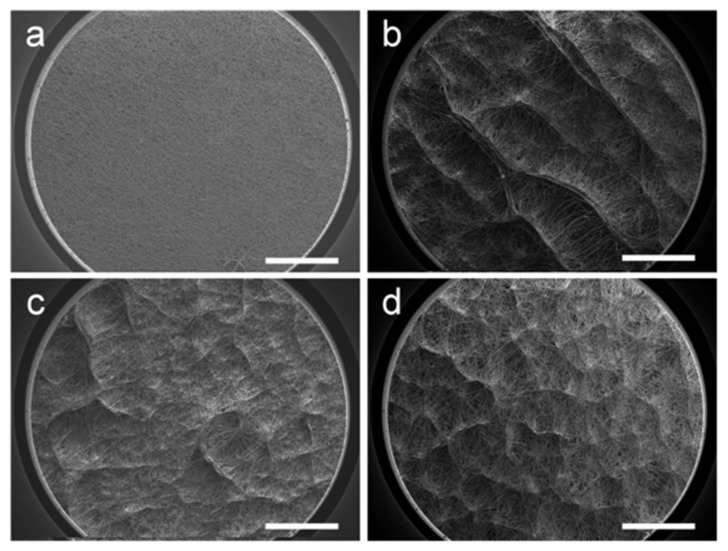
SEM images of e-spun films at different receiving temperatures—(**a**) 23 °C, (**b**) 0 °C, (**c**) −10 °C and (**d**) −20 °C—at a humidity of 50%. Scale bars are 1 mm.

Varying patterns of the honeycomb structure were formed by altering the processing parameters. To investigate the influence of temperature, the working distance between the needle and collector was set at 15 cm and the humidity was kept at 50%. The results are presented in Figure 3. As shown in Figure 3a, the surface of the e-spun membrane obtained at room temperature is relatively flat, and there is no obvious surface topology. Moreover, the fiber arrangement is random and uniform. However, all three samples in low-temperature (0 °C, −10 °C, and −20 °C) conditions (Figure 3b–d) displayed obvious three-dimensional structures. This uneven macroscopic topography is formed by the self-assembly of fibers, which have raised surface structures and interconnected pores. It is worth noting that the wall structure at 0 °C is more continuous and pronounced, and the wall is thicker. The macrostructure at −10 °C becomes thin and scattered. The self-assembled macrostructures are uniformly distributed on the surface of the fiber membrane. When the receiving temperature is lowered to −20 °C, the shape of the macrostructure is more similar to that of the −10 °C sample, but the number of raised structures formed by the parallel arrangement of fibers increases and the fiber membrane becomes fluffy. It can be inferred that the cold air above the surface of the receiving plate affects the orientation of the fiber in this interval. With heat exchange, there is also a low-temperature room above the cryogenic substrate. Being different from that in room-temperature conditions, the fiber movement during e-spinning in the low-temperature room is disturbed, making it sensitive to residual charges in the filaments previously deposited on the collector, resulting in an uneven macrostructure.

**Figure 4:**
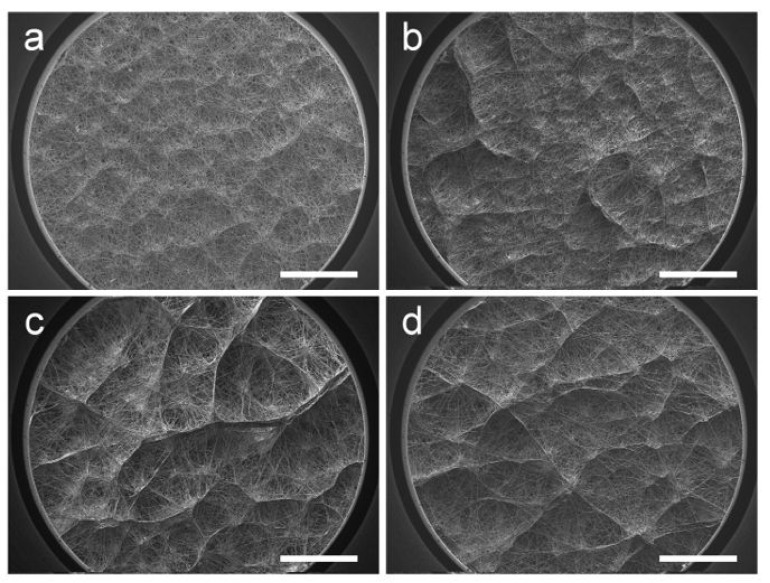
SEM images of e-spun films at different humidity levels—(**a**) 40%, (**b**) 50%, (**c**) 60% and (**d**) 70%—at a receiving temperature of −10 °C. Scale bars are 1 mm.

During the experiment, when the receiving plate temperature dropped below 0 °C, a thin layer of ice crystals appeared, making the nearby water in the air solidify [15]. In addition, the existence of ice crystals likely affected the arrangement and direction of the fibers, resulting in the difference in macro morphology. In order to deeply explore the influence of ice crystals and humidity, we set four moisture gradients. When investigating the influence of humidity, the working distance between the needle and the collector was set at 15 cm and the receiving temperature was kept at −10 °C. The difference is shown in Figure 4. As can be observed, the macro pores of the 40% humidity sample are few and sparse. Moreover, the honeycomb-like structures become distinct when the humidity is over 60%. Higher humidity suppresses volatilization of the solvent, so the pre-deposited filaments carry more residual charges, which has proven to be relevant to the onset of honeycomb structures. Furthermore, when the humidity was high, large ice crystals were able to form among the deposited e-spun filaments. These ice crystals were subsequently sublimated and wiped out during lyophilization, so the e-spun filaments dangled (Figure 4c,d). In conclusion, both decreasing the receiving temperature and increasing the humidity induced the formation of a macroscopic uneven structure.

**Figure 5:**
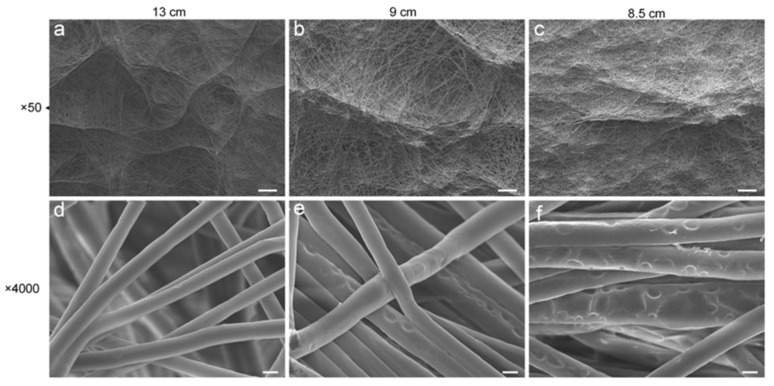
SEM images at different collecting distances during cryogenic e-spinning in high humidity (**a**–**f**). The receiving temperature was −10 °C and the ambient humidity was 70%. Scale bars are 200 μm (**a**–**c**) and 2 μm (**d**–**f**).

According to published reports, the rough surface of the macrostructure has an inducing effect on cell growth [16]; the microporous structures on the fiber surface entice cells to adhere and proliferate [17]. However, there is no report on the utilization of cryogenic electrospinning technology to obtain self-assembled fiber membranes with macroscopic uneven structures, and no fiber membrane with both self-assembled macrostructures and microstructures on the fiber surface has been obtained. Here, by controlling the collecting distance, microporous filaments were acquired when maintaining the macrostructures (Figure 5). In this set of experiments, in order to suppress solvent volatilization as much as possible, to obtain micropores on the fiber surface [15], an ambient humidity of 70% was selected. As the collecting distance decreases, it can be observed that the filaments become more porous, while the overall macrostructure is still maintained. For the macroscopic wall-like structure, the shape of the honeycomb structures at a receiving distance of 13 cm (Figure 5a) is pronounced, and the structures are connected to each other. As the distance decreases (Figure 5b,c), the raised wall-like structures become thicker in shape and sparsely distributed on the e-spun membrane. Due to shortening of the fiber acceptance distance, the fiber cannot be fully stretched in the air, and the macroscopic uneven morphology obtained at this time is inhibited, to a certain extent. The macrostructures at this time (Figure 5c) are more similar to sparsely distributed raised structures than regular honeycomb patterns. The honeycomb alignment cannot be observed uniformly across the surface. Another major effect of the reduced receiving distance is the change in fiber diameter and the creation of surface micropores (Figure 5d–f). The fiber diameters at receiving distances of 13 cm, 9 cm and 8.5 cm are 2.15 ± 0.20 μm, 2.76 ± 0.45 μm and 3.01 ± 0.58 μm, respectively. Shortening of the receiving distance leads to shortening of the period during which the fiber is stretched by the electric field force, which tends to make the fiber become thicker. At the same time, as the receiving distance becomes shorter, the micropores on the fiber surface are gradually generated and increased. Volatilization of the solvent in the fiber is suppressed when the fiber flying distance is short. Under this condition, the residual solvent in the deposited fiber is influenced by the cryogenic template, phase separation occurs and the solvent aggregates on the fiber surface. After freeze-drying, the residual solvent on the fiber surface formed into small ice crystals and were eliminated; thus, the microporous filament appeared [15]. 

**Figure 6:**
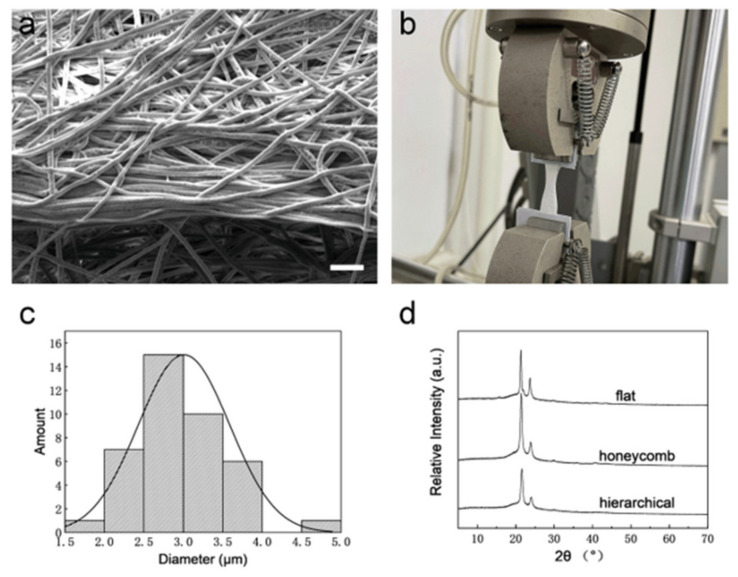
Characterization of the hierarchical self-assembled film (obtained at a receiving plate temperature of −10 °C, an ambient humidity of 70% and a fiber receiving distance of 8.5 cm). (**a**) SEM image of hierarchical structures self-assembled by uniform porous fibers when the collecting distance is 8.5 cm. The scale bar is 20 μm. (**b**) Mechanical test. (**c**) Fiber diameter distribution. (**d**) XRD patterns of flat film, honeycomb film (which has a self-assembled structure, but smooth fiber surface) and hierarchical film (which has a self-assembled structure and micropores on the fiber surface) (the flat film sample was obtained with a receiving temperature of 23 °C, an ambient humidity of 50% and a fiber acceptance distance of 15 cm. The honeycomb film sample was obtained with a receiving temperature of −10 °C, an ambient humidity of 50% and a fiber acceptance distance of 15 cm. The hierarchical film sample was obtained with a receiving temperature of −10 °C, an ambient humidity of 70% and a fiber acceptance distance of 8.5 cm).

Here, the e-spun membrane obtained at a receiving plate temperature of −10 °C, an ambient humidity of 70% and a fiber receiving distance of 8.5 cm was chosen as the hierarchical self-assembly film (Figure 6a), which has a uniform fiber diameter and hierarchical structures. The hierarchically structured fiber membrane has both macroscopic structures composed of fiber self-assembly on the membrane surface and microporous structures induced by phase separation on the filament surface. Meanwhile, the mechanical properties of the hierarchical self-assembly film were tested (Figure 6b). In order to test the influence of structure anisotropy on the mechanical properties, uneven mesh was cut into two rectangle pieces; then, one piece was stretched along the parallel direction of the wall, while the other piece was stretched along the vertical direction of the wall. As a result, the parallel piece displayed a better Young’s modulus and sustained a higher force before fracture, indicating that the wall-like structures of e-spun film reinforce mechanical properties (Table 1). Thus, anisotropic uneven constructs have potential applications for the repair of organs with an anisotropic structure, such as the esophagus [18]. Ultrafine filament with a uniform fiber diameter is an important prerequisite for the effective mechanical properties of fiber membranes [19]. During the stretching process of the e-spun film, we found that it is more likely to break in areas where the material is thinner or of poorer quality. Therefore, continuous ultrafine fibers without a bead-like structure or breakpoint ensure the stability of the mechanical properties of the membrane. Compared with the bimodal fiber diameters in traditional self-assembly e-spun film [20], the fiber diameters of cryogenic e-spun film in this study are more homogeneous, ranging from 1.5 to 5 μm (Figure 6c). Moreover, no bead-like fiber was observed in the SEM tests. The XRD patterns are shown in Figure 6d. The flat film sample was obtained with a receiving temperature of 23 °C, an ambient humidity of 50% and a fiber acceptance distance of 15 cm. The honeycomb film sample (which had a self-assembled structure, but smooth fiber surface) was obtained with a receiving temperature of −10 °C, an ambient humidity of 50% and a fiber acceptance distance of 15 cm. The hierarchical film sample (which had a self-assembled structure and micropores on the fiber surface) was obtained with a receiving temperature of −10 °C, an ambient humidity of 70% and a fiber acceptance distance of 8.5 cm. The typical peaks of PCL (2θ = 21.7° and 24°) were found in all three samples, which indicates that e-spun mesh with different morphologies have similar crystalline structures. Moreover, the full width at half maximum (FWHM) of the flat film at both peaks was narrower than that of the other two films, suggesting that cryogenic e-spinning decreases crystallinity. 

**Figure 7:**
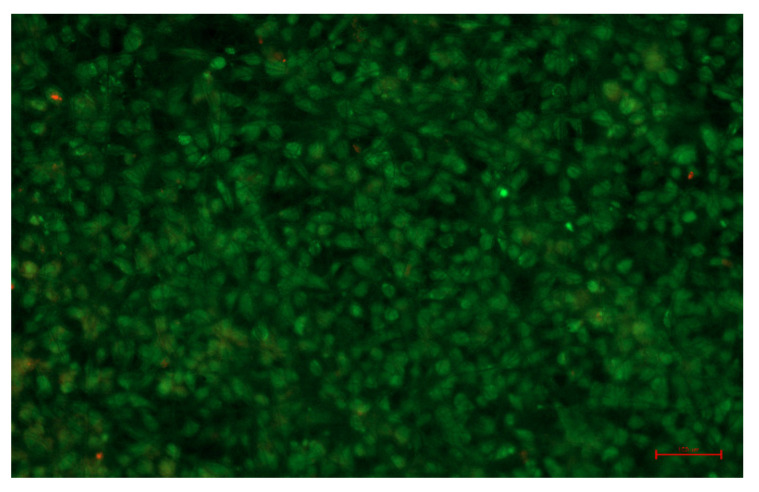
SHEE cell viability on the e-spun hierarchical film. The scale bar is 100 μm.

The acquisition of hierarchically structured fibrous membranes demonstrates the great potential of template-free cryogenic electrospinning technology for the preparation of tissue engineering membranes. The self-assembled fibrous membrane with a hierarchical structure displays anisotropic mechanical properties and a topology similar to that of the esophageal mucosa. Here, the effect of this fibrous membrane on esophageal epithelial cells was assessed and in vitro cell experiments were performed. The cell viability test is shown in Figure 7. Esophageal epithelial cells proliferate massively on the e-spun membrane, and the cells are closely arranged, displaying excellent cell viability. These results prove the promising potential applications for esophageal repair [21].

## 4. Conclusions

In this work, cryogenic e-spinning was adopted to fabricate the self-assembly of homogeneous e-spun fibers. We found that a uniform fiber diameter ensures excellent mechanical properties. The wall-like structure obtained through self-assembly creates anisotropy and reinforces the mechanical properties for e-spun films. In addition, micropores were created on the e-spun fiber surface, providing a hierarchical structure. Finally, esophageal epithelial cells grow well on the cryogenic e-spun fibers, which demonstrates that the obtained uneven films can be used in esophagus repair.

## Figures and Tables

**Table 1:** Anisotropy of hierarchical film with uneven morphology (obtained with a receiving plate temperature of −10 °C, an ambient humidity of 70% and a fiber receiving distance of 8.5 cm).

	Parameter	Young’s Modulus (MPa)	Maximum Load (N)
Group			
Parallel		2.13 ± 0.46	3.09 ± 0.23
Vertical		1.10 ± 0.11	1.41 ± 0.51

## Data Availability

The data presented in this study are available on request from the corresponding author.
